# Mini review on the synthesis of porous hydroxyapatite nanomaterials for dental tissue engineering

**DOI:** 10.1039/d5ra08770h

**Published:** 2026-05-28

**Authors:** Alexandr Zibert, Sascha Balakin, Ankit Mazumdar, Garcia-Zintzun Aidee I, Ika Dewi Ana, Natalia Beshchasna, Joerg Opitz

**Affiliations:** a Fraunhofer Institute for Ceramic Technologies and Systems IKTS Germany alexandr.zibert@ikts.fraunhofer.de; b Dental Biomedical Sciences Department, Faculty of Dentistry, Universitas Gadjah Mada Jalan Denta No. 1 Sekip Utara Yogyakarta 55281 Indonesia; c Center of Excellence for Carbonate Apatite-based Extracellular Matrix and Adjuvant, Integrated Research and Testing Laboratory (LPPT), Universitas Gadjah Mada Yogyakarta 55281 Indonesia

## Abstract

Hydroxyapatite is a ceramic material composed of calcium phosphate, the most abundant mineral in human bone and teeth. Its physical and chemical properties allow its applications in bone tissue engineering, particularly in dental tissue engineering. Nanostructured hydroxyapatite is not widely used for biomedical applications yet, but it is now of great interest to the scientific community. This work summarises different ways for the synthesis of hydroxyapatite nanostructures having versatile morphologies, with an emphasis on wet chemical methods, and shows the possible applications of hydroxyapatite in dental applications and dental tissue engineering.

## Abstract 2

This review summarises current wet chemical synthesis methods for hydroxyapatite nano- and micro-structures and explores their applications in dentistry and dental tissue engineering.

## Graphical abstract

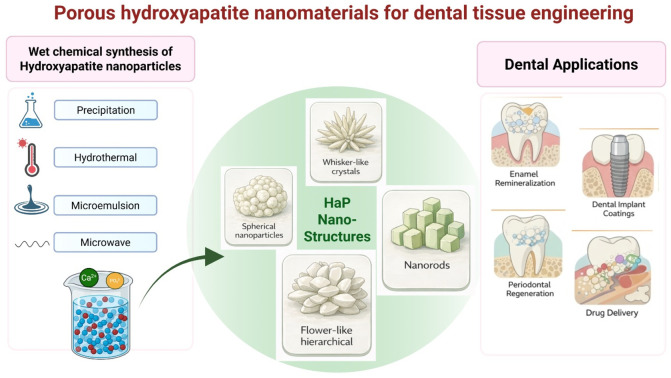

## 1 Introduction

Tissue engineering (TE) in stomatology enables the reconstruction and regeneration of damaged tissues using synthetic materials, which maintain an osteogenic microenvironment, high biocompatibility, and similar mechanical properties to those of natural tissues.^1^ In addition to the conscientious material design, the constrains imposed dictated by nature play a significant role. The oral cavity provides a complex situation where bacteria-driven diseases such as periodontitis, pulpitis, and cavities damage dental tissues.^2^ The adequate regeneration of periodontal tissues damaged by such diseases requires the harmonic growth of soft and hard tissues.^3^ In this regard, the guided tissue/bone regeneration method is a promising pathway. This method uses membranes as scaffolds to form new periodontal ligament attachments.^3^ Different types of membranes based on polymers (*e.g.*, tetrafluoroethylene) and metals (titanium and cobalt)^4^ have been developed. Despite the benefits of polymeric membranes, such as low antigenicity, high biocompatibility, and excellent cell affinity, they have some limitations—they are less suited for alveolar bone regeneration, lack rigidity, have the tendency to collapse due to poor mechanical properties and can be colonized by bacteria. Enhanced biological and mechanical properties of scaffolds and membranes used in dental TE can be achieved by incorporating or grafting them with functional nanomaterials that can exhibit therapeutic, restorative, or antimicrobial properties.^5^ In order to engineer superior materials specifically for dental applications, the TE triad concept, comprising a combination of progenitor cells and scaffold and growth factors, utilizes the interplay of biology, chemistry, and materials science for the design process. Bioactive molecules and the employed materials influence the human body's response at cellular and systemic levels.^6^ Besides enhancing the mechanical properties and the biocompatibility of the scaffold, nanomaterials facilitate augmented functionalities in combination with bioactive molecules. Growth factors, nucleic acids, and endocrine hormones administered *via* nanomaterials alter cell differentiation and proliferation. On the other hand, the nanomaterial itself can show antibacterial properties or accelerate cell proliferation.^7,8^

Recently, porous hydroxyapatite (HAP) nanomaterials have been investigated due to their availability, low cost, simple production methods, bioactivity and biocompatibility. HAP nanomaterials are prominent candidates in bone TE due to their chemical and physical similarities with human bone.^9^ Moreover, HAP is widely used in orthopedics, dentistry, cancer treatment, drug delivery, and implant coating, where its chemical content and morphology significantly affect the resulting properties and future applications.^10^ The application of HAP ceramics for implants meets the ASTM F 1185-88 standard. Polymeric scaffolds containing HAP nanomaterials provide better mechanical properties and a lower degradation rate compared to bare polylactic or collagen/chitosan scaffolds.^10–12^ Compared to the synthesis of other materials, such as tricalcium phosphate and mineral trioxide aggregates, HAP synthesis is inexpensive, and the morphology of the final HAP nanomaterial can be adjusted according to clinical requirements. Due to its hexagonal lattice, HAP presents elongated nanoparticles.^13^ However, various morphologies are achievable, such as nanorods, spherical nanomaterials, and hollow or porous structures, based on the synthesis pathways.^9^

**Fig. 1:**
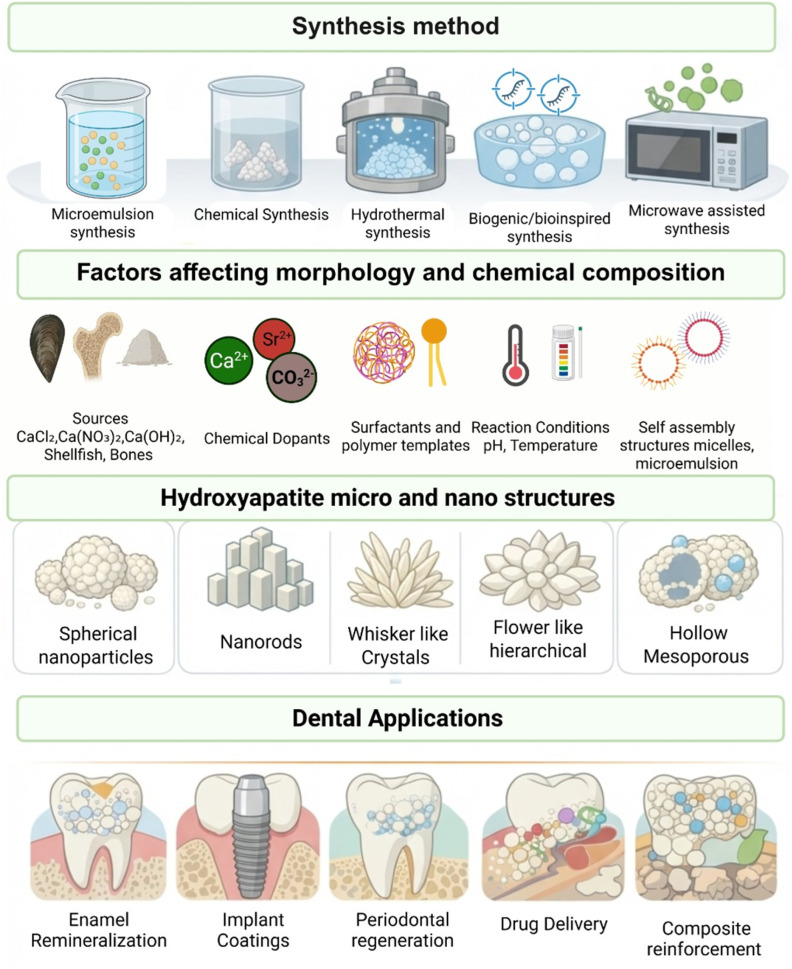
Graphical representation of the different wet chemical synthesis pathways.

On that account, porous HAP nanomaterials are gaining tremendous attention, especially for use in complex biological environments, due to their inherent combination of unique properties. Combining porous morphologies with their osteoconductive properties, HAP nanomaterials pave the way for personalized medicines with fewer side effects, sustainable local drug delivery, and high biocompatibility. Therefore, this mini review summarizes several synthesis methods of HAP nanomaterials, with a focus on their dental applications (Fig. 1).

## 2. Wet chemical synthesis and modification methods of porous HAP nanomaterials

The synthesis of HAP nanomaterials includes dry, wet, and high temperature methods, synthesis from biogenic sources, and combination procedures. In this review, focus will be placed on the wet chemical method and the combination method due to their robustness, effective synthesis control and raw material availability.^14^Table 1 summarizes the wet chemical-based fabrication methods of porous HAP nanomaterials and their properties.

**Table 1:** Summary of the wet chemical routes for HAP synthesis

Fabrication method	HAP size/shape	Bioactive additives	Key findings/effects reported	Bioactivity/biocompatibility	References
Chemical precipitation method	35–59 nm doped HAP nanoparticles	Metal ions incorporated into the structure	Incorporation of metal ions into the structure leads to an antibacterial effect	High level of haemocompatibility	15
Microwave-assisted wet chemical method	Au-loaded HAP particles with immobilized collagen	Doxorubicin	Pro-osteogenic effects and tunable release characteristics	Significant cytotoxicity, in case of use in scaffolds, excellent cellular attachment, growth and proliferation	16
Microemulsion method	Predominantly spherical structures, high purity, magnetic HAP	Fe	Good control of a product morphology by altering the synthesis conditions	Addition of magnetic properties, high cell proliferation rate	17 and 18
Chemical precipitation of HAP derived from biogenic sources	High crystallinity µm-sized particles	Sr^2+^ ions	Higher crystallinity, Ca:P stochiometric ratio is more similar to the natural bone	Better viability of pre-osteoblast cells. Sr^2+^ dopants are expected to improve osteoconductivity	19
Opposite ion core–shell method	3–4 µm hollow porous spheres	Ibuprofen (model)	Not complicated synthesis, possibility to use for implant grafting and drug delivery	No additional information	20
Hydrothermal	Core–shell HAP structures, 7 µm in diameter	N/A	Synthesized microspheres can be used in drug delivery	N/A	21

There are several techniques used to produce HAP nanoparticles using the wet chemical method: chemical precipitation, microwave-assisted wet chemical method, inverse microemulsion method, sonochemistry-assisted microwave method, opposite ion core–shell method, and microwave-assisted method. The chemical precipitation method is interesting due to its simplicity and low cost, providing good control for morphology and shape, but the resultant nanoparticles' crystallinity is less compared to other methods.^22,23^ The reverse microemulsion method is simple and gives better control over the shape of the resultant particles and allows the production of structures with different morphologies (hollow nanoparticles and multilayered nanoparticles).^24^ The opposite ion core–shell method allows the simple production of hollow nanostructures with a porous morphology.^20^ Microwave-assisted and sonochemistry-assisted microwave methods yield highly crystalline, pure, and fine particles and are often used to synthesize HAP composite nanoparticles (Mondal *et al.*, 2019).^16^ The hydrothermal method allows the production of HAP micro- and nano-structures with high crystallinity, good control over size and diverse morphologies (nanoparticles, nanoplatelets, and nanorods).^25^

In order to modify the porous HAP nanomaterials for enhanced therapeutic effects, they can be loaded with growth factors or antimicrobial agents. Furthermore, doping ions can alter the properties of HAP. The incorporation of Sr^2+^ and Bi^3+^ ions results in antimicrobial properties, and such particles were synthesized with a microwave-assisted method; alternatively, substitution with Mg^2+^ and Sr^2+^ results in enhanced cell attachment, proliferation, and differentiation of fibroblast cells.^26^ For instance, the inclusion of Ag^+^ provides antimicrobial properties, and together with Zn^2+^, Mg^2+^ and Sr^2+^, it supports bone formation;^27^ it has been previously reported that doping glass-reinforced hydroxyapatite composites with Sm^3+^ improves osteoblastic cell response and antibacterial effects.^28,29^

Additionally, it is of great interest to have a porous HAP for the delivery and controlled release of bioactive proteins, such as BMP-2 growth factor.^14,30,31^ The additional benefit of using HAP is in enhancing the mechanical properties of the membranes.

### 2.1 Chemical precipitation

The chemical precipitation method is widely used due to its scalability and relatively low cost. This method usually includes the mixing of a Ca^2+^ source and a PO_4_^3−^ source in stoichiometric concentrations, followed by adjusting the pH with further filtering, washing, drying, and grinding the precipitate. The typical outcome is particles with low to moderate crystallinity and a broad size distribution. Due to the low energy consumption and the requirement of only simple equipment, this method is easily scalable. Yet, the limitation of this method is the poor control over morphology or the low crystallinity of the obtained particles in comparison with other methods.^32^

### 2.2 Opposite ion core–shell method

The opposite ion core–shell method is a “hard template method”. Vaterite, a metastable polymorph of calcite, nanoparticles are used as a template for the deposition of mesoporous HAP. Vaterite nanoparticles are synthesized by the chemical precipitation method, similar to the formation of calcite nanoparticles, but by changing the reaction media, reaction kinetics, and nucleation rate, reaction yields spherical mesoporous vaterite crystals, defining the morphology of HAP. After chemical deposition, vaterite nanoparticles are treated with phosphoric acid to get a porous hydroxyapatite shell by replacing the CO_3_^2−^ ions of vaterite with PO_4_^3−^ ions. Next, the as prepared core–shell particles are exposed to acetic acid solution, and due to the difference in the solubility rates of hydroxyapatite and vaterite, such a reaction would yield only hollow mesoporous HAP particles.^33^ This method allows a relatively simple (sometimes one-pot) deposition of HAP nanoparticles with a hollow and mesoporous morphology. Yet, due to the low crystallinity of the vaterite template, the size of the particles is usually of the micrometer range and the size distribution is wide.

Hollow porous hydroxyapatite spheres are interesting due to the possibility of encapsulating drugs. The large particle size potentially allows encapsulating larger molecules, such as proteins, which could be beneficial for the delivery of growth factors, such as BMP-2. Some studies have reported encapsulating ibuprofen, vancomycin hydrochloride, and ciprofloxacin hydrochloride in the as-prepared structures,^34,35^ which allows the application of such structures for local drug delivery in peri-implantitis treatment or post-surgical care.

### 2.3 Inverse microemulsion method

This method is based on the creation of a water-in-oil emulsion with nanosized water droplets, stabilized by surfactants and co-surfactants, acting as reactors for the particle growth. Confining the reaction environment helps in overcoming agglomeration during synthesis and provides better control over morphology and crystallinity, even in cases when the lattice is doped. Adjusting water and oil ratios helps in adjusting the size.^36^ The method allows to yield particles with a uniform size and does not require sophisticated equipment, but it requires organic solvents, such as cyclohexane, making it less environmentally friendly. Control over size and morphology is crucial in applications requiring a high surface area or small particle size, such as in composite glass ionomer cement (GIC) for filling enamel cavities.

### 2.4 Hydrothermal method

The hydrothermal method is based on increasing the reactivity of precursors using high temperature and pressure. In comparison with chemical precipitation, and while the same or similar precursors can be used (*e.g.* calcium hydroxide as the Ca^2+^ source and orthophosphoric acid as the PO_4_^3−^ source),^22^ it allows the production of nanoparticles with higher crystallinity. The uniform distribution of particle size can be achieved by using surfactants, such as CTAB (cetrimonium bromide), during synthesis. On the other hand, this method is more demanding: it requires an autoclave or a pressure vessel as the equipment and consumes more energy. Moreover, surfactants can be added to obtain porous structures *via* this method as it is reported to synthesize rod-like nanomaterials of 15–20 nm width, 60–70 nm length, and 2–10 nm pore size.^21^ The increased crystallinity allows the doping or co-precipitation of nanoparticles with different elements. As an example, it is^37^ reported in the literature that the synthesis of a TiO_2_-HAP nanocomposite, which exhibits antibacterial properties and could be used for the whitening and remineralization of enamel.

### 2.5 Microwave-assisted method

The synthesis of HAP by the microwave-assisted method is based on the microwave radiation in the range of 700–900 W. It allows the production of 20–30 nm-sized HAP nanoparticles incorporated with Si, Mg, Sr, or F. Researches have shown that HAP powders obtained using this technique are more similar to natural bone, and due to the presence of substituted ions, they exhibit better bioactive properties. It was reported that for this type of synthesis, even a simple microwave oven (Sharp R728 K) could be used. This approach offers the advantages of a precipitation method, such as low energy consumption, robustness, and low price, and yields particles with a better crystallinity and finer size.^38–40^ One of the main challenges of this method is its scalability. In large vessels of several hundred millilitre capacities, an uneven distribution of the electromagnetic field inside the reactor might lead to a broad size particle distribution and poor reproducibility of results.

### 2.6 Biogenic chemical precipitation method

One of the emerging green synthesis routes for HAP NP synthesis is from Ca-rich biogenic sources, such as corals, eggshells, mussel shells, fish scales or animal bones. This direction has gained extensive attention due to the possibility of performing environmentally friendly synthesis and achieving HAP NPs with enhanced properties. In general, there are a variety of HAP synthesis methods using biogenic sources, which include solid state synthesis, microwave-assisted synthesis, sol–gel method, and hydrothermal synthesis.^41^ Some of them allow the yield of HAP NPs directly; for example, alkaline or thermal treatment followed by the grinding of fish scales or bones would remove the organic compounds from the structure and yield HAP NPs. In this case, the properties of the resultant HAP NPs will be dependent on the donor species. Several studies report the application of HAP NPs derived from tilapia fish scales in dentistry and investigate their impact on orthodontic tooth movement and periodontal ligament space.^42^

The other direction is the application of Ca-rich biogenic sources, such as the exoskeletons of mollusks or marine species, to obtain a solution of Ca salts for further co-precipitation. This method preserves the benefits of biogenic synthesis and provides better control of the nanoparticle structure and chemical composition.^19^

HAP nanoparticles produced by this method contain trace ions which are contained in natural apatite, enhancing the biocompatibility and bioactivity even further, which not only supports the green synthesis strategy but also might boost its performance in orthodontic tooth movement modulation, periodontal regeneration and enamel remineralization. Yet, the intrinsic composition of biogenic sources strongly depends on the species of the donor, its age, habitat, and diseases, which leads to problems in reproducibility, complicating the clinical scalability of this method.

### 2.7 Chemical routes to influence the morphology and content of HAP nanoparticles

**Fig. 2:**
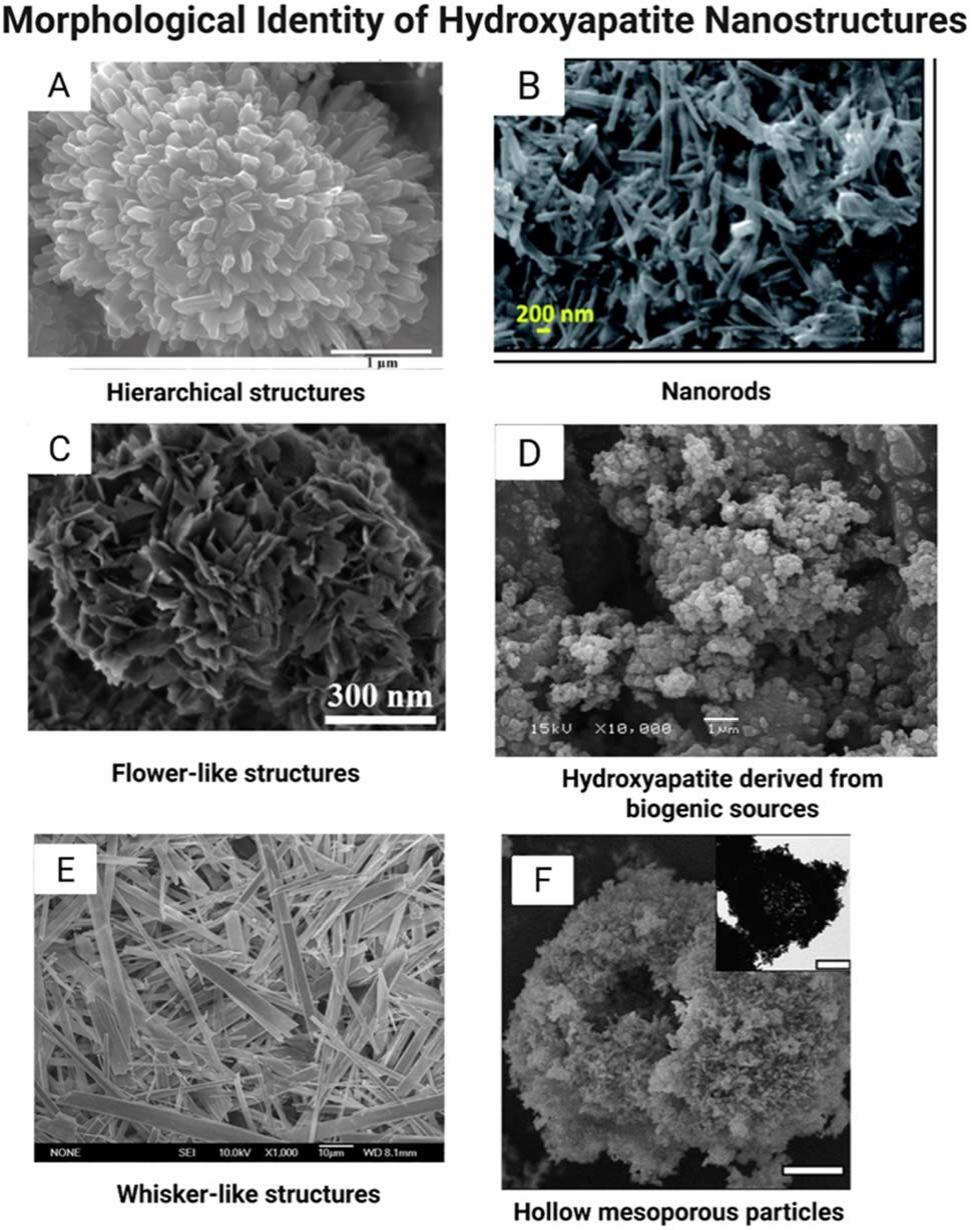
SEM and TEM images of hydroxyapatite structures with different morphologies, as adapted from the literature. (A) Reproduced from ref. 45, Elsevier, licensed under CC BY 4.0. (B) Reproduced from ref. 46 with permission from the Royal Society of Chemistry. (C) Reproduced from ref. 47 with permission from the Royal Society of Chemistry. (D) Reproduced from ref. 48 with permission from the Royal Society of Chemistry. (E) Reproduced from ref. 49 with permission from the Royal Society of Chemistry. (F) Reproduced from ref. 50 with permission from the Royal Society of Chemistry.

The methods described above could be modified further by altering the reaction media *via* the choice of different solvents or the application of surfactants, co-surfactants, templates (synthetic or biogenic), nanogels, polymeric matrices, organic molecules, adsorbates, crystal growth modifiers, ionic substituents, chelating agents or biogenic additives. Fig. 2 shows examples of the influence of a combination of the chosen synthesis routes and reaction parameters. These changes could alter the kinetics of the reaction, provide nucleation sites, affect crystal growth, change nucleation rate, or introduce other elements to the lattice, which would affect the resultant morphology and/or chemical composition, leading to a change in the biological response. One of the examples is the biomimetic approach: if a charged polymer (PVA, gelatin) is introduced into the system during the precipitation of HAP, positively charged Ca^2+^ ions will first be bonded to negatively charged moieties, which will be followed by the attraction of phosphate anions. This method allows a more precise control of the size distribution and agglomeration and yields HAP that structurally resembles HAP in the natural bone.^43^ Another example is the modulation of the morphology of HAP nanorods using fluoride. In a recent study, Meng *et al.*^44^ synthesized fluoride-doped HAP nanorods *via* a hydrothermal method and showed that fluoride alters the orientation and size of crystallites, and they studied the *in vivo* efficiency of these nanorods in enamel remineralization.

## 3 Dental applications

HAP is a main component of tooth enamel and is abundant in dentine. This, combined with properties such as non-toxicity, biocompatibility, bioactivity, osteoconductivity, and good tissue adhesion, makes it an ideal candidate to be used for dental applications. On the other hand, in the case of applications in dental tissue engineering, porous HAP nanoparticles can be used since they can act as nanocarriers of bioactive molecules.^51,52^

### 3.1 Enamel remineralization for caries treatment

The early stage of caries can be described by enamel demineralization, mostly due to a change in pH caused by nutrition or the presence of metabolic products of bacteria on teeth. Tooth enamel is composed of 20–40 nm-sized HAP nanoparticles. The application of synthetic HAP nanoparticles can cause the remineralization of enamel and decrease its demineralization by providing a source of calcium and phosphate ions. HAP nanoparticles could also fill the gaps in tooth enamel, preventing bacterial growth and thus decreasing the rate of demineralization, act as a natural whitening agent and be used in early tooth decay treatment.^53^ HAP NPs for these purposes can be derived from natural sources, such as abalone mussel shell, combined with carbomer as a carrier.^41^ An advantage of HAP over fluoride here is its better performance in acidic environments, which determines the better performance in treating oral cavity. It also helps to avoid the risk of long exposure of tissues to fluoride compounds, which could lead to fluorosis. In the research^54^ authors have performed *in vitro* research on 60 extracted human teeth using SEM and EDX analysis, which confirmed the remineralization of enamel using commercial-HAP-containing toothpastes after teeth were demineralized. Another research^55^ have shown that HAP nanoparticles substituted with four different ions (Mg, Zn, Sr, or Si) performed better in remineralization compared to pure HAP.

### 3.2 Coating of dental implants

Implants for dental applications should meet several criteria, such as the desired physical and chemical properties, thermal stability, biocompatibility, and mechanical properties such as flexural strength, high elasticity modulus, and impact strength. Due to the mechanical properties of HAP, implants cannot be made of this material. On the other hand, metallic implants, for example, made of stainless steel, titanium, or cobalt-chrome alloys, meet the desired mechanical properties but lack biocompatibility. This issue is overcome by using HAP as a coating of metallic implants, using techniques such as pulsed laser deposition coating, electrophoretic deposition coating, thermal spraying, sol gel or other methods, improving new bone growth, bone bonding and bone-to-implant bonding.^56,57^ The advantages of HAP over other materials used for coating are its biocompatibility and bioactivity. TiO_2_ films or coatings provide better mechanical support but lack adhesion to the bone, while calcium phosphates (close to the HAP group of materials) are less stable in the oral cavity media. For example, one study ^58^ has shown that Ti implants coated with HAP implanted into ovariectomized rats exhibited a higher reverse torque, implant-to-bone contact and formation of new bone tissues. Another research^59^ has shown that HAP coatings reduce biofilm formation in comparison with uncoated implants, pointing out to its antibacterial properties and the potential to prevent the development of peri-implantitis (Table 2).

**Table 2:** Summary of the dental applications of HAP

Type of application	Function of nHAP	HAP composition	Application method	Advantages	Limitations	Notes/effectiveness
Enamel remineralization (early caries, prophylaxis)	Mineral source; enamel defect filling; antibacterial properties (if doped)	Pure or doped nHAP, 20–100 nm	Component of toothpastes, gels or polishing pastes	Avoids fluoride-related risks (fluorosis); low abrasiveness	Limited penetration depth; mainly effective in early lesions	Remineralization comparable to fluoride-containing formulations^60^
Coating of dental implants	Biocompatible interface between the metal and tissue	nHAP of various sizes and morphologies	Plasma spraying, templating, sol–gel, electrophoretic or electrostatic spray, as a coating or composite layer	Biomimicry, biocompatibility, osseointegration, enhanced early bone bonding, possibility to introduce antibacterial properties	Brittleness, susceptibility to cracking, and limited adhesion to metals	Higher implant stability quotient, reduced risk of peri-implantitis, higher bone-implant contact^61^
nHAP-GIC composites	Mechanical reinforcement; improved bioactivity	nHAP, 10–200 nm, various morphologies	Used as a composite filler in GIC	Enhanced ion exchange and fluoride release; improved biocompatibility	Strong dependence on particle size and morphology	Higher compressive and flexural strength, enhanced mineralization, reduced microleakage^62^

### 3.3 Nanohydroxyapatite composites for glass ionomer cement

Glass ionomer cement (GIC) is widely used in dentistry as a restorative material due to properties such as good adhesion to the teeth tissue, low coefficient of thermal expansion, calcium and fluoride ion release, and biocompatibility. Nevertheless, its clinical applications are limited by low fracture strength, poor stress resistance, and high sensitivity to moisture; GIC composites with additive materials are used to overcome these limitations. One of the materials used as an additive is hydroxyapatite. HAP can be implemented into the GIC structure by the chemical precipitation method, hydrothermal method, or by a solid state reaction. Usually, more than one material is included in GIC composites, and HAP is used due to its stability in biological media, biocompatibility and mechanical properties. Studies have shown that the incorporation of HAP into GIC composition improves mechanical properties, resulting in an increase in compressive strength by 113.6 MPa, an increase in fracture toughness, an improvement in microhardness properties and a significant increase in flexural strength.^63^ Tuygunov *et al.* have formulated a GIC composite with HAP and tetracalcium phosphate (TTCP).^64^ Their study has shown that HAP increases compressive strength, while TTCP reduces mechanical strength, and such formulations can meet ISO criteria for restorative applications.

## 4 Future aspects

Some studies present the methods of achieving both porous and doped hydroxyapatite microspheres by adding a solution of a salt of desired elements to the Ca ion source.^65–68^ New directions in this field could be the application of HAP nanoparticles for the functionalization or biofunctionalization of tooth enamel or dental implant coatings by incorporating HAP NP-containing payloads with desired biological properties, such as enhanced biological adhesion or antibacterial properties. Another direction might be the application of AI in modelling the physical or chemical properties of HAP NPs or the synthesis parameters. Lastly, it is important to develop a means for robust and scaled-up production of such materials for the possibility of being applied on an industrial scale.

## 5 Conclusions

This work has summarized several methods for the synthesis of different types of HAP nanoparticles and their composites with different physical, chemical, and biological properties. These properties, combined with the vast possibilities of HAP modifications, make this material a versatile instrument in bone tissue engineering and dentistry, providing a platform for preventive, regenerative, antibacterial, and restorative dental therapies and thereby addressing clinical needs such as enhancing the biological properties of scaffolds for bone and dental tissue engineering, oral cavity restoration and the prevention of related diseases.

## Author contributions

Alexandr Zibert: writing – original draft, investigation, review and editing, Sascha Balakin: supervision, review and editing, conceptualization. Ankit Mazumdar: visualization, Garcia-Zintzun Aidee I: review and editing, Ika Dewi Ana: investigation, review and editing, Natalia Beshchasna: project administration, supervision, Jörg Opitz: project administration, supervision.

## Conflicts of interest

There are no conflicts to declare.

## Data Availability

No primary research results, software or code have been included and no new data were generated or analysed as part of this review. All data used in this minireview were obtained from previously published sources, which are cited within the text. No new datasets were generated or analyzed during this study.
